# Cardiac Rehabilitation Increases SIRT1 Activity and *β*-Hydroxybutyrate Levels and Decreases Oxidative Stress in Patients with HF with Preserved Ejection Fraction

**DOI:** 10.1155/2019/7049237

**Published:** 2019-11-27

**Authors:** Graziamaria Corbi, Valeria Conti, Jacopo Troisi, Angelo Colucci, Valentina Manzo, Paola Di Pietro, Maria Consiglia Calabrese, Albino Carrizzo, Carmine Vecchione, Nicola Ferrara, Amelia Filippelli

**Affiliations:** ^1^Department of Medicine and Health Sciences, University of Molise, Campobasso, Italy; ^2^Department of Medicine, Surgery and Dentistry, University of Salerno, Baronissi, Italy; ^3^Theoreo srl, Via degli Ulivi 3 84090 Montecorvino Pugliano, Italy; ^4^European Biomedical Research Institute of Salerno (EBRIS), Via S. de Renzi 3, 84125 Salerno, Italy; ^5^IRCCS Neuromed, Department of Vascular Physiopathology, Pozzilli, Italy; ^6^Department of Translational Medical Sciences, Federico II University of Naples, Naples, Italy; ^7^Istituti Clinici Scientifici Maugeri SpA Società Benefit (ICS Maugeri SpA SB), Telese Terme, Italy

## Abstract

**Purpose:**

Exercise training induces beneficial effects also by increasing levels of Sirtuin 1 (Sirt1) and *β*-hydroxybutyrate (*β*OHB). Up to date, no studies investigated the role of exercise training-based cardiac rehabilitation (ET-CR) programs on *β*OHB levels. Therefore, the present study is aimed at investigating whether a supervised 4-week ET-CR program was able to induce changes in Sirt1 activity and *β*OHB levels and to evaluate the possible relationship between such parameters, in Heart Failure with preserved Ejection Fraction (HFpEF) patients.

**Methods:**

A prospective longitudinal observational study was conducted on patients consecutively admitted to the Cardiology and Cardiac Rehabilitation Units of “San Gennaro dei Poveri” Hospital in Naples, Italy. In fifty elderly patients affected by HFpEF, in NYHA II and III class, Sirt1 activity, Trolox Equivalent Antioxidant Capacity (TEAC), *β*OHB, and Oxidized Low-Density Lipoprotein (Ox-LDL) levels were measured before and at the end of the ET-CR program. A control group of 20 HFpEF patients was also recruited, and the same parameters were evaluated 4 weeks after the beginning of the study.

**Results:**

ET-CR induced an increase of Sirt1 activity, *β*OHB levels, and antioxidant capacity. Moreover, it was associated with a rise in NAD^+^ and NAD^+^/NADH ratio levels and a reduction in Ox-LDL. No changes affected the controls.

**Conclusion:**

The characterization of the ET-CR effects from a metabolic viewpoint might represent an important step to improve the HFpEF management.

## 1. Introduction

Despite recent advances in both pharmacological and nonpharmacological therapies, heart failure (HF) is still a prevalent cause of death or permanent invalidity worldwide [1]. The exercise training-based cardiac rehabilitation (ET-CR) surely represents a valid nonpharmacological therapeutic approach against HF; nevertheless, it is still underprescribed in aged patients. The reason for this behavior could be ascribed to their comorbidity and polytherapy that complicate the participation in the ET-CR programs.

In HF, tissue hypoxia, caused either by low cardiac output or by sympathetic vasoconstriction, may also trigger a significant increase in the production of free radicals [2]. In fact, oxidative stress, which occurs when reactive oxygen species (ROS) are produced in excess and overcome the action of the endogenous antioxidants mechanisms, is implicated in the pathophysiology of HF. This is proved by a correlation between oxidative stress markers and HF in human and animal studies [3, 4] and by direct molecular evidence about an etiological role of ROS [5] in cardiovascular diseases, including HF.

During life, the cardiovascular system is constantly exposed to oxidative stress; hence, the balance between the production of ROS and activation of the antioxidant defence system is crucial for the human physiology and control of the cellular homeostasis [6].

Several *in vitro* and *in vivo* studies have demonstrated that ROS activation might occur in HF as a response to various stressors [7]; animal studies have also suggested that antioxidants and ROS defence pathways can ameliorate ROS-mediated cardiac abnormalities [8].

Up to date, no effective therapies for reducing morbidity or mortality in HF with preserved ejection fraction (HFpEF) are available, limiting the treatment for symptom relief and comorbidity management in such a category of patients [9, 10]. A key barrier to therapeutic development is a significant lack of knowledge about HFpEF pathogenesis and pathophysiology [11, 12]. Thus, elucidating molecular mechanisms and identifying novel therapeutic targets in the HFpEF phenotype are essential needs to improve the management of these patients [13].

A recent meta-analysis demonstrated that ET-CR is associated with improvements in cardiorespiratory fitness and quality of life of the patients with HFpEF [14]. ET, as part of CR, is effective in inducing beneficial effects at cardiac level via the reduction of the oxidant amount and stimulation of the antioxidant capacity [15, 16].

An important mechanism, involved in the cellular response to exogenous stressors, is represented by the sirtuins, NAD^+^-dependent deacetylases, now recognized as oxidative stress sensors and modulators of cellular redox state [17, 18].

A supervised ET-CR program increases the activity of the best-characterized member of sirtuins, Sirt1. As a consequence, a systemic antioxidant defence in elderly HFpEF patients is stimulated by inducing the activation of Sirt1's molecular targets, such as the antioxidants superoxide dismutases (SODs) and catalase [19]. Interestingly, Nagao et al. [20] have demonstrated that myocardial *β*OHB has the potential to exert compensatory antioxidant effects under pathological conditions. In particular, the authors found that *β*OHB was elevated in failing mouse hearts, attenuated ROS production, and alleviated apoptosis induced by oxidative stress, suggesting that a build-up of *β*OHB might occur as a compensatory response against oxidative stress in failing hearts. Besides, ketone bodies have been proposed as agents mimicking the effects of caloric restriction which is considered a valid therapeutic approach linked to the beneficial effects of Sirt1 [21]. So far, no studies have been performed to investigate the role of an ET-CR program on *β*OHB levels. Therefore, the main aims of the present study were to investigate whether a supervised 4-week ET-CR program was able to induce changes in Sirt1 activity and *β*OHB levels and to evaluate the possible relationship between these two parameters in HFpEF patients.

## 2. Methods

### 2.1. Study Design and Population

A prospective longitudinal observational study was conducted in patients consecutively admitted to the Cardiology and Cardiac Rehabilitation Units of “San Gennaro dei Poveri” Hospital of Naples, Italy. Patients' written informed consent forms were collected; the study was approved by the local Medical Research Ethics Committee and was performed in accordance with the Declaration of Helsinki Fifth Revision (2013) and its amendments. This report adheres to the standards for the reporting of observational trials and was written according to the STROBE guidelines for Observational Studies in Epidemiology-Molecular Epidemiology (STROBE-ME) [22].

Male elderly subjects with HF in clinically stable condition, classified as in NYHA II and III class and with a preserved ejection fraction (EF) (70 with HF preserved EF), were enrolled. All definitions were based on the ESC and ACCF/AHA criteria, in which the term “stable” defines treated patients with symptoms and signs remained generally unchanged for at least a month [23, 24].

Of the study population, 50 patients underwent a well-structured ET-CR program of 4 weeks, while 20 patients represented the control group. The reasons why the control group did not undergo ET-CR program were related to individual circumstances that have made unpractical the participation in an outpatient program (e.g., patients who lived in a long-term care facility or no cardiac rehabilitation program available within 60 minutes of travel time from the patient's home).

The exclusion criteria included unstable angina pectoris, use of nitrates, uncompensated HF, complex ventricular arrhythmias, pacemaker implantation, and orthopedic or neurological limitations to exercise. No sex-based or racial/ethnic-based differences were present between the groups.

All enrolled patients underwent a physical examination, collection of demographic and routine blood chemistry tests, chest X-ray, blood pressure measurement, electrocardiographic and echocardiographic examinations, and a cardiopulmonary stress test at baseline.

After 4 weeks, both groups underwent physical examination and blood chemistry tests.

None of the patients had experienced a myocardial infarction in the 12 months preceding the study, and based on body mass index, none were cachectic (Table 1).

### 2.2. Training Protocol

Patients underwent a 4-week structured exercise training, on a hospital ambulatory-based regimen. At an initial stage, on a cycle ergometer, the progression of aerobic exercise training provided an intensity set at 50% VO2 max, based on the performance achieved in the cardiopulmonary stress test. The exercise duration was increased from 15 to 30 min, according to perceived symptoms and clinical status, for the first 1–2 weeks. A gradual increase of intensity (60–70% of peak VO2, if tolerated) was achieved within 2 weeks [25]. The target of 60–70% VO2 peak was then utilized to schedule each exercise session at the beginning of the 4-week training program. The exercise workload was gradually increased until the achievement of the predefined target. Each session was forerun by a 10 min unloaded warm-up phase and followed by a 5 min unloaded cool-down [26]. The training sessions were performed 5 times per week, under continuous electrocardiographic monitoring, and supervised by a cardiologist, a physiotherapist, and a graduate nurse.

### 2.3. Blood Sample Collection

Overnight fasting blood samples were obtained at baseline and after 4 weeks in both the groups. After centrifugation at 1500 × *g* for 10 min, plasma samples were transferred to new tubes and stored at -80°C until analysis. Peripheral blood mononuclear cells (PBMCs) were isolated from whole blood by Ficoll-Paque PLUS (GE Healthcare, Munich, Germany), according to the manufacturer's procedures.

### 2.4. Sirt1 Activity

Sirt1 activity was determined, in nuclei extracted by PBMCs of all recruited subjects, using a SIRT1/Sir2 Deacetylase Fluorometric Assay (CycLex, Ina, Nagano, Japan) and 96 flat bottom transparent polystyrene plates (Thermo Fisher Scientific, USA), following the manufacturer's instructions. Values were reported as relative fluorescence/*μ*g of protein (AU). All data are expressed as the mean ± SD of three independent experiments. Replicated sample analysis showed a coefficient of variation (CV) < 5%.

### 2.5. *β*-Hydroxybutyrate Plasma Levels

*β*-Hydroxybutyrate (*β*OHB) extraction, purification, and derivatization were carried by the MetaboPrep GC kit (Theoreo, Montecorvino Pugliano, Italy). According to the protocol by Troisi et al. [27], 50 *μ*L of sample was added to 200 *μ*L of extraction mix solution containing the internal standard. The sample and extraction mixture were vortexing at 1250 rpm for 30 seconds. The extract was centrifuged for 5 minutes at 16000 rpm, keeping the temperature below 4°C. Two hundred microliters of the upper liquid phase was removed and transferred into a microcentrifuge tube containing the purification mixture (200 *μ*L). This was vortexed at 1250 rpm for 30 seconds. A rapid centrifuge of the sample (to prevent the sediment suspension) at 16000 rpm was performed keeping the temperature below 4°C. One hundred seventy-five microliters of liquid upper phase was transferred into the glass vial and freeze-dried overnight.

After the derivatization, the extract was transferred in a 100 *μ*L insert for the autosampler injection. This was centrifuged for 5 minutes at 16000 rpm keeping the temperature below 4°C before injecting. The sample (2 *μ*L) was analyzed using a gas chromatography-mass spectrometry (GC-MS) system (GC-2010 Plus gas chromatography coupled to a 2010 Plus single quadrupole mass spectrometer; Shimadzu Corp., Kyoto, Japan). Chromatographic separation was achieved with a 30 m 0.25 mm CP-Sil 8 CB fused silica capillary GC column with 1.00 *μ*m film thickness from Agilent (Agilent, J&W), with helium as the carrier gas.

*β*OHB was evaluated quantitatively by the use of external calibration. The analytical standard was purchased from Sigma-Aldrich (Milan, Italy). Five calibration standards were prepared, freeze-dried overnight to eliminate the solvent, and derivatized with the same procedure of the samples. GC-MS *β*OHB calibration curve showed an *R*^2^ = 0.997, while replicated samples analysis showed a coefficient of variation (CV) < 10% and the analytical standard was analyzed in triplicate.

### 2.6. Oxidative Stress Markers

Total antioxidant capacity (Trolox Equivalent Antioxidant Capacity (TEAC)) and Oxidized Low-Density Lipoproteins (Ox-LDL) were measured in plasma samples isolated from the patients who underwent the ET-CR and the controls. The TEAC assay was performed according to the protocol already described in the authors' previous study [28].

The levels of Ox-LDL were determined, by using a human Ox-LDL ELISA Kit (MyBiosource, Inc., USA) and 96 flat bottom transparent polystyrene plates (Thermo Fisher Scientific, USA), following the manufacturer's instructions.

### 2.7. NAD^+^/NADH Ratio

NAD^+^/NADH ratio was quantified using the EnzyCromTM NAD^+^/NADH Assay Kit with a detection limit of 0.05 microM and linearity up to 10 microM (BioAssay Systems, Hayward, CA) and 96 flat bottom transparent polystyrene plates (Thermo Fisher Scientific, USA), following the manufacturer's instructions. The optical density was read at 565 nm at time zero (OD0) and, after incubation (15 min), at room temperature (OD15). OD values were used to determine the NAD^+^/NADH concentration of each sample from a standard curve. All data are expressed as the mean ± SD of three independent experiments.

### 2.8. Statistical Analysis

Continuous variables are expressed as the mean ± standard deviation compared with paired or unpaired Student's *t*-test (normally distributed variables), or as median ± interquartile range value compared with the Mann-Whitney *U* test (not normally distributed). Normality of data distribution was evaluated using the Kolmogorov-Smirnov test. Nonnormally distributed continuous variables were converted to their natural log functions. Categorical variables are expressed as a proportion and compared with the *χ*^2^ test.

Correlation between variables were assessed by linear regression analysis, and variables, which demonstrated statistical significance in a univariate model, were then included in a multivariate analysis. All data were analyzed using SPSS version 23.0 (SPSS, Inc., Chicago, Illinois, USA). Statistical significance was accepted at *p* < 0.05.

## 3. Results

The study population consisted of 70 male subjects (mean age 69.5 ± 4.27 years) affected by HFpEF. All patients completed the study. At baseline, no differences in medical therapy were found between the groups, and no therapeutic changes occurred during the study period (Table 1).

Table 1 shows the main demographic, hemodynamic, and chemical characteristics of the group who underwent a 4-week ET-CR program and the control group. Changes in some hemodynamic variables after 4 weeks are reported in Table 2. The ET-CR was able to induce a significant reduction in systolic blood pressure and an increase in ejection fraction. No changes were observed in the controls (Table 2).

**Figure 1:**
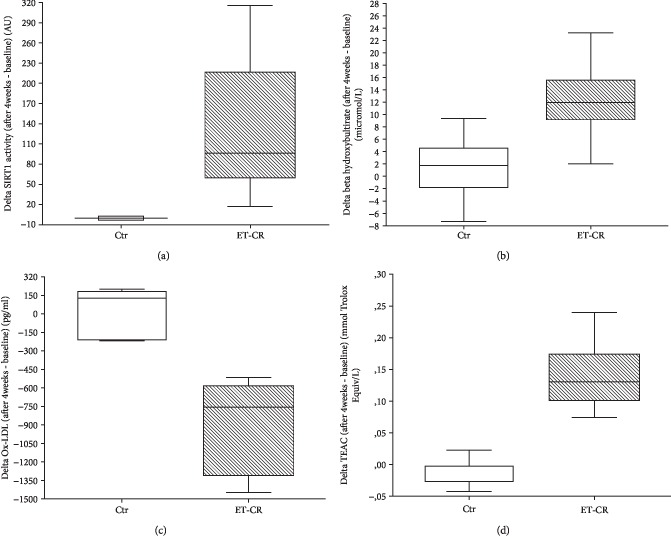
Changes in control and ET-CR groups of Sirt1 activity, *β*-hydroxybutyrate, Ox-LDL levels, and TEAC from baseline to 4 weeks after the study start. The ET-CR was able to induce a significant increase in Sirt1 activity (a) and *β*-hydroxybutyrate (*β*OHB) (b) (both, *p* < 0.0001); a reduction in Ox-LDL (c) (*p* < 0.001) and increased levels of antioxidant response measured by TEAC assay (d) (*p* < 0.0001), as showed by the difference between the levels after 4 weeks minus the levels at baseline.

Table 3 and Figure 1 show the changes in Sirt1 activity, *β*OHB, and oxidant and antioxidant parameters, at baseline and after 4 weeks. No differences were found between the groups at baseline, while significant differences were found between groups and intragroup (see above). The ET-CR induced a significant increase in Sirt1 activity, *β*OHB, and antioxidant capacity measured by TEAC assay, as shown by the raised levels of such parameters in the ET-CR group but not in the controls (Table 3 and Figures 1(a), 1(b), and 1(d)) and decreased levels of Ox-LDL in the ET-CR group but not in the controls (Figure 1(c)).

**Figure 2:**
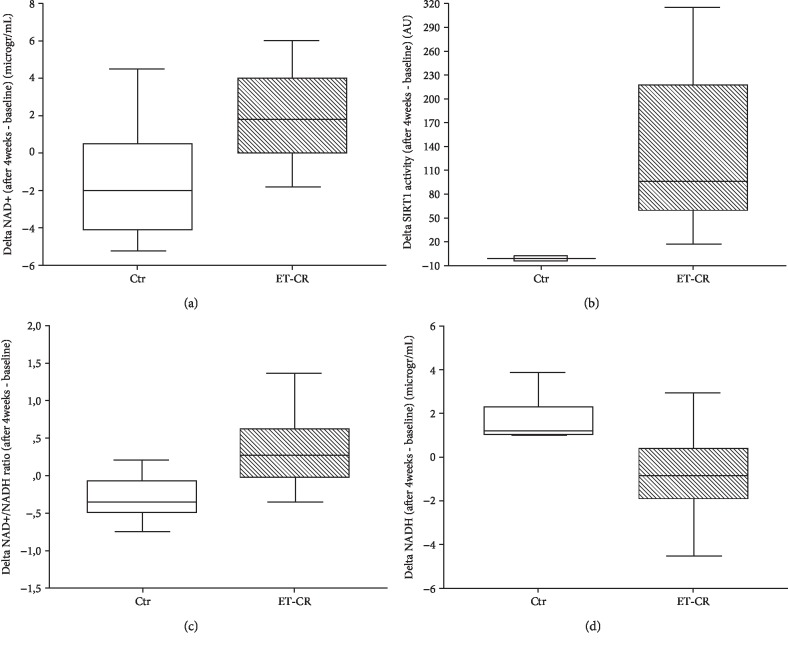
Changes in the control and ET-CR groups of Sirt1 activity, NAD^+^, NADH levels, and NAD^+^/NADH ratio from baseline to 4 weeks after the study start. The ET-CR was able to induce a significant increase in NAD^+^ (a), Sirt1 activity (b), and NAD^+^/NADH ratio (c) (all *p* < 0.0001), associated with a reduction in NADH levels (d) (*p* < 0.001), expressed as the difference between the levels after 4 weeks minus the levels at baseline.

Moreover, the ET-CR was effective in inducing a significant increase in NAD^+^ and NAD^+^/NADH ratio and a decrease in NADH (all *p* < 0.0001, Table 3 and Figures 2(a), 2(c), and 2(d)), while no changes were found in the controls.

**Figure 3:**
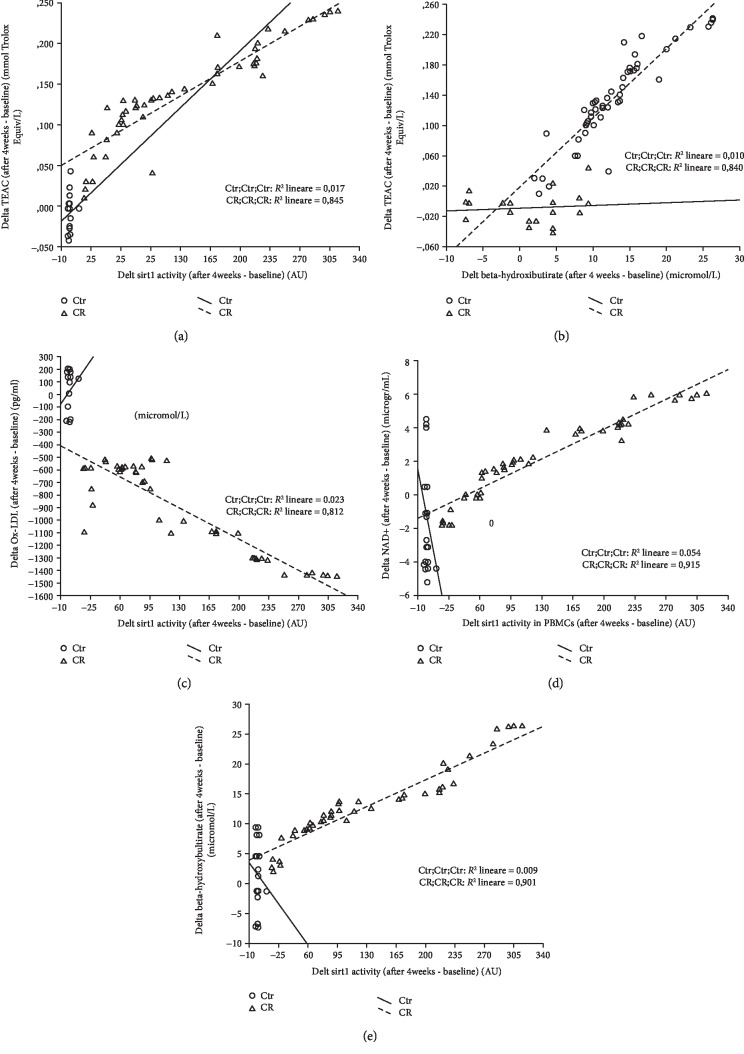
Linear regression correlation among delta Sirt1 activity and delta oxidants and antioxidants. (a) Linear regression correlation between TEAC and delta Sirt1 activity. (b) Linear regression correlation between delta TEAC and delta *β*OHB levels. (c) Linear regression correlation between delta Ox-LDL and delta Sirt1 activity. (d) Linear regression correlation between delta NAD^+^ and delta Sirt1 activity. (e) Linear regression correlation between delta *β*OHB levels and delta Sirt1 activity.

All these findings were confirmed when all the parameters were expressed as differences between levels after 4 weeks minus baseline levels (delta, Figures 1–3). Notably, the increasing delta levels of NAD^+^ and NAD^+^/NADH ratio were associated with increasing levels of delta Sirt1 activity (Figures 2(a)–2(c)), as expected by the requirement of NAD^+^ for Sirt1 activity.

By a multivariate linear regression analysis, introducing the delta TEAC as a dependent variable, we found that the best predictors of the changes in antioxidant levels were represented by the delta Sirt1 activity (*p* < 0.0001, *r*^2^ = 0.845; *β* = 0.000; 95% CI 0.000–0.001; Figure 3(a)) and the ET-CR group (*p* < 0.0001, *β* = 0.061; 95% CI 0.045–0.076) followed by the delta *β*OHB levels (*p* = 0.032, *r*^2^ = 0.840; *β* = 0.002; 95% CI 0.000–0.004; Figure 3(b)). Moreover, introducing the delta Ox-LDL as dependent variable, we found that the best predictors of the oxidant levels changes were represented by the delta Sirt1 activity (*p* < 0.0001, *r*^2^ = 0.812; *β* = −5.639; 95% CI -6.839 to -4.438; Figure 3(c)) and the ET-CR group (*p* < 0.0001, *β* = −510.5; 95% CI -614.3 to -406.7). In particular, higher changes in TEAC and Ox-LDL were associated with higher changes in SIRT1 activity in a direct (*r*^2^ = 0.845, Figure 3(a)) and inverse relationship (*r*^2^ = 0.812, Figure 3(c)), respectively.

Finally, introducing in a multivariate linear regression analysis the delta NAD^+^ as a dependent variable, we found that the best predictors were the delta Sirt1 activity (*p* < 0.0001, *r*^2^ = 0.915; *β* = 0.051; 95% CI 0.037–0.065; Figure 3(d)), followed by the ET-CR group (*p* = 0.004, *β* = 2.71; 95% CI 0.920–4.494).

A strong direct association was found between the delta Sirt1 activity and the delta of NAD^+^ levels (*r*^2^ = 0.915, Figure 3(d)) and between the delta of *β*OHB levels and the delta of Sirt1 activity (*r*^2^ = 0.901, Figure 3(e)).

## 4. Discussion

In the present study, we have demonstrated that a well-structured 4-week ET-CR program was able to increase the levels of Sirt1 activity and *β*-hydroxybutyrate, and these findings were associated with an improvement of TEAC and a reduction of Ox-LDL.

To treat and especially manage the patients with HFpEF can be very challenging. This is mostly caused by a significant lack of knowledge in this field. For this reason, there is now high interest to elucidate the pathophysiology of the different HF phenotypes.

From a molecular point of view, the ET-CR might represent not only a valuable complementary therapeutic approach but also a study model to expose the molecular actors involved in HFpEF.

Previously, we demonstrated that Sirt1 was able to mediate the ET-CR effects at a molecular level inducing activation of its target catalase [19].

Several studies have demonstrated that both Sirt1 and *β*OHB are involved in the antioxidant cellular response. In particular, increased circulating levels of *β*OHB were linked to a reduction in oxidative stress [29], increased AMPK activity [30], and autophagy [31].

Moreover, *β*OHB was found to be an endogenous inhibitor of class I and IIa histone deacetylases (HDACs) [32] but not of the sirtuins (class III HDACs) representing a structurally distinct group of NAD-dependent deacetylases, in which *β*OHB is not known to directly regulate [33].

*β*OHB seems to work as a mimetic of caloric restriction that is the most known natural activator of some sirtuins [21]. Edwards et al. [34] have demonstrated that a *β*OHB administration in *C. elegans* delayed glucose toxicity and extended the worm's lifespan in a Sir2- (the homolog of the human Sirt1) dependent manner. Therefore, these authors have proposed *β*OHB as a valuable treatment against aging-associated disorders [34].

Similar to the caloric restriction, the exercise training (ET) is recognized as a helpful tool against cardiovascular diseases. Indeed, ET is widely recommended in HF for its beneficial effects on the exercise tolerance [35].

Noteworthy, both caloric restriction and ET are associated with a significant increase of ketone bodies, such as *β*OHB [36–38].

Although the metabolic profiles differ in dependence on the timing and duration of physical activity, both short-term and long-term studies have sought to characterize the biochemical response to exercise [36]. Interestingly, Matoulek et al. showed that *β*OHB increased after exercise in patients who underwent a three-month fitness program [39].

Moreover, an acute bout of aerobic exercise increases class IIa HDAC phosphorylation and subsequent nuclear exclusion, thus inhibiting HDAC-mediated repression of specific exercise-responsive genes such as GLUT4 and PGC-1*α* [40, 41]. This suggests that compounds such as *β*OHB could be used to mimic or enhance adaptations to a physical exercise [36].

In our study, an ET-CR program was able to induce, in patients affected by HFpEF, an increase of both Sirt1 and *β*OHB, in association to a better antioxidant activity, as showed by higher levels of TEAC and NAD^+^.

Notably, it has been proposed that the relative sparing of cytoplasmic NAD levels with the utilization of *β*OHB, rather than glucose, can alter the activity of NAD-dependent enzymes such as sirtuins [42]. Recent studies observed an increase in the mitochondrial *β*-hydroxybutyrate dehydrogenase (BDH1), which coincided with elevated plasma levels of *β*OHB in both rodent and human models of heart failure [43, 44]. Increased amount of *β*OHB oxidation in isolated perfused hearts was also found [43]. These studies suggested that the increase of ketone body metabolism could represent an additional strategy leading to a metabolic remodeling in the failing heart. However, whether this is an adaptive or maladaptive response remains uncertain [45]. In this context, to better characterize the ET-CR effects from the metabolic point of view can represent an important step to improve the HFpEF management.

### 4.1. Limitations

A possible limitation of this study is the lack of cell sorting useful to identify what cells compose the PBMCs that could be mainly involved in the observed molecular modifications. However, Sirt1 is a ubiquitous molecule and several studies have shown changes in Sirt1 activity in PBMCs without a distinction of the PBMC cell types [28, 46].

Another limitation could be the lack of women in the study population. We did recruit only three women and then decided to exclude them because of the small number. This is in line with the fact that women are less inclined to take part in exercise training-based cardiac rehabilitation programs [47, 48]. Therefore, further studies are necessary to better clarify the molecular effects of ET-CR also in female patients.

## 5. Conclusions

The ability of exercise training to regulate metabolic and oxidative stress response can explain why ET-CR can be considered a sort of pharmacological tool in CVD management. In particular, ET-CR is a helpful medical practice in which several molecular factors mutually influence each other. The exercise training included in CR programs acts as a nonpharmacological inductor of antioxidant response. The HFpEF represents a peculiar phenotype of HF whose pathophysiological aspects have yet to be clarified.

Further studies should be addressed to evaluate the role of ET-CR in influencing the evolution of HFpEF considering the molecular changes induced by this tool to better clarify the mechanism and the pathway involved in the genesis and progression of the disease.

## Figures and Tables

**Table 1:** Main characteristics of total population and ET-CR group at baseline.

Variables	Total population	Ctr	ET-CR	*p*
Age (years), mean ± SD	69.5 ± 4.3	70.25 ± 4.7	69.20 ± 4.1	0.357
BMI (kg/m^2^), mean ± SD	27.6 ± 3.2	26.7 ± 3.3	27.9 ± 3.1	0.154
SBP (mmHg), mean ± SD	120.9 ± 11.0	119.3 ± 11.0	121.5 ± 11.0	0.443
DBP (mmHg), mean ± SD	71.7 ± 5.7	71.0 ± 5.3	72.0 ± 5.9	0.511
EF (%), mean ± SD	56.7 ± 4.0	57.9 ± 3.8	56.2 ± 4.0	0.117
LVEDD (mm)	52.27 ± 4.27	52.95 ± 4.19	52.00 ± 4.35	0.404
CAD, *n* (%)	14 (71.4)	14 (70)	36 (72)	0.542
PTCA, *n* (%)	37 (52.9)	11 (55)	26 (52)	0.516
CABG, *n* (%)	10 (14.3)	3 (15)	7 (14)	0.59
Previous IMA, *n* (%)	47 (67.1)	13 (65)	(68)	0.51
Valvular substitution, *n* (%)	3 (4.3)	1 (5)	2 (4)	0.642
Smoking, *n* (%)	37 (52.9)	9 (45)	28 (56)	0.285
Hypertension, *n* (%)	30 (42.9)	8 (40)	22 (44)	0.487
Dislipidemia, *n* (%)	31 (44.3)	9 (45)	22 (44)	0.574
Diabetes, *n* (%)	14 (20)	4 (20)	10 (20)	0.619
COPD, *n* (%)	13 (18.6)	4 (20)	9 (18)	0.545
Beta blockers	64 (91.4)	18 (90)	46 (92)	0.556
ACE inhibitors	32 (45.7)	9 (45)	23 (46)	0.576
ARBs	9 (12.9)	2 (10)	7 (14)	0.495
Diuretics	20 (28.6)	5 (25)	15 (30)	0.458
Ca2 antagonists	7 (10)	2 (10)	5 (10)	0.652
Aspirin	56 (80)	15 (75)	41 (82)	0.361
Anticoagulants	33 (47.1)	9 (45)	24 (48)	0.516
Oral hypoglycemics	11 (15.7)	4 (20)	7 (14)	0.385
Insulin	5 (7.1)	1 (5)	4 (8)	0.556
Statin	53 (75.7)	15 (75)	38 (76)	0.578

Data are expressed as the mean ± SD or number of subjects (%). BMI: body mass index; SBP: systolic blood pressure; DPB: diastolic blood pressure; EF: ejection fraction; LVEDD: left end diastolic diameter; CAD: coronary artery disease; PTCA: percutaneous transluminal coronary angioplasty; CABG: coronary artery bypass graft; COPD: chronic obstructive pulmonary disease; ARBs: angiotensin II receptor blockers.

**Table 2:** Changes in some hemodynamic variables in HFpEF controls and HFpEF patients who underwent ET-CR.

Variables	Ctr		*p*	ET-CR		*p*
	Baseline	After 4 weeks		Baseline	After 4 weeks	
SBP (mmHg), mean ± SD	119.3 ± 11.0	119.75 ± 10.6	0.163	121.5 ± 11.0	120.26 ± 8.8	**0.026**
DBP (mmHg), mean ± SD	71.0 ± 5.3	71.5 ± 5.2	0.163	72.0 ± 5.9	71.8 ± 5.3	0.159
EF (%), mean ± SD	57.9 ± 3.8	57.6 ± 3.5	0.110	56.2 ± 4.0	57.22 ± 3.19	**0.001**
LVEDD (mm)	52.95 ± 4.19	53.15 ± 4.23	0.428	52.0 ± 4.35	51.86 ± 3.96	0.442

SBP: systolic blood pressure; DPB: diastolic blood pressure; EF: ejection fraction; LVEDD: left end diastolic diameter.

**Table 3:** Changes in oxidant/antioxidant parameters in HFpEF controls and HFpEF patients who underwent ET-CR.

Variables	Ctr		*p*	ET-CR		*p*
	Baseline	After 4 weeks		Baseline	After 4 weeks	
SIRT1 activity (AU)	1941.80 ± 149.35	1942.16 ± 149.67	0.560	1953.14 ± 125.06	2082.44 ± 108.68^∗^	<0.0001
*β*OHB (*μ*mol/L)	47.96 ± 4.84	49.35 ± 5.21	0.280	48.76 ± 3.27	61.58 ± 6.91^∗^	<0.0001
Ox-LDL (pg/mL)	3227.62 ± 281.13	3255.33 ± 388.57	0.492	3286.49 ± 527.08	2380.47 ± 608.30^∗^	<0.0001
TEAC (mmol Trolox Equiv/L)	0.295 ± 0.084	0.286 ± 0.722	0.075	0.290 ± 0.723	0.425 ± 0.061^∗^	<0.0001
NAD^+^	19.74 ± 2.03	18.41 ± 2.49	0.136	19.50 ± 1.48	21.64 ± 1.27^∗^	<0.0001
NADH	12.26 ± 1.43	13.25 ± 1.67	0.090	12.16 ± 0.97	11.15 ± 1.09^∗^	<0.001
NAD^+^/NADH	1.64 ± 0.32	1.44 ± 0.40	0.115	1.62 ± 0.24	1.96 ± 0.28^∗^	<0.0001

ET-CR: exercise training-based cardiac rehabilitation; *β*OHB: *β*-hydroxybutyrate; Ox-LDL: Oxidized Low-Density Lipoprotein. ^∗^CR vs. Ctr after 4 weeks, *p* < 0.0001.

## Data Availability

The data used to support the findings of this study are available from the corresponding author upon request.
